# What Maintains the Central North Pacific Genetic Discontinuity in Pacific Herring?

**DOI:** 10.1371/journal.pone.0050340

**Published:** 2012-12-28

**Authors:** Ming Liu, Longshan Lin, Tianxiang Gao, Takashi Yanagimoto, Yasunori Sakurai, W. Stewart Grant

**Affiliations:** 1 Institute of Evolution and Marine Biodiversity, Ocean University of China, Qingdao, Shandong, China; 2 National Research Institute of Fisheries Science, Fisheries Research Agency, Yokohama, Kanagawa, Japan; 3 Graduate School of Fisheries Science, Hokkaido University, Hakodate, Hokkaido, Japan; 4 Alaska Department of Fish and Game, Anchorage, Alaska, United States of America; Biodiversity Insitute of Ontario – University of Guelph, Canada

## Abstract

Pacific herring show an abrupt genetic discontinuity in the central North Pacific that represents secondary contact between refuge populations previously isolated during Pleistocene glaciations. Paradoxically, high levels of gene flow produce genetic homogeneity among ocean-type populations within each group. Here, we surveyed variability in mtDNA control-region sequences (463 bp) and nine microsatellite loci in Pacific herring from sites across the North Pacific to further explore the nature of the genetic discontinuity around the Alaska Peninsula. Consistent with previous studies, little divergence (Φ_ST_  = 0.011) was detected between ocean-type populations of Pacific herring in the North West Pacific, except for a population in the Yellow Sea (Φ_ST_  = 0.065). A moderate reduction in genetic diversity for both mtDNA and microsatellites in the Yellow Sea likely reflects founder effects during the last colonization of this sea. Reciprocal monophyly between divergent mtDNA lineages (Φ_ST_  = 0.391) across the Alaska Peninsula defines the discontinuity across the North Pacific. However, microsatellites did not show a strong break, as eastern Bering Sea (EBS) herring were more closely related to NE Pacific than to NW Pacific herring. This discordance between mtDNA and microsatellites may be due to microsatellite allelic convergence or to sex-biased dispersal across the secondary contact zone. The sharp discontinuity between Pacific herring populations may be maintained by high-density blocking, competitive exclusion or hybrid inferiority.

## Introduction

Untangling the influences that historical events and present-day processes have on phylogeographic patterns in marine species is a major focus of phylogeographic research [1]. Genetic studies show a wide range of shallow population structure that can often be related to shoreline complexity [2], ocean frontal systems [3], retentive or dispersive currents [4], [5], or intrinsic biological limits on dispersal [6], [7]. Marine species are often imprinted with deep structure from historical isolations. Among these are examples of abrupt phylogeographic breaks along a shoreline that appear to be unrelated to contemporary barriers to gene flow [8]–[10]. These population discontinuities are not always concordant with general biogeographic breaks observed in other species, and thus appear to reflect historical demography [11] or interactions between populations [12]. The extent that population boundaries reflect interactions between divergent populations within a species has not been examined in depth.

Here, we focus on a deep geographic partition between populations of Pacific herring in the central North Pacific that has previously been detected with allozyme [13], [14] and mitochondrial (mt) DNA markers [15], [16]. Three divergent mtDNA lineages appear in Pacific herring: one is largely restricted to the Northwestern (NW) Pacific and Bering Sea, and the other two are restricted to the Northeastern (NE) Pacific. Ocean-type herring, which complete their life cycle in high salinity water [14], tend to show little divergence among populations within these two groups [13], [14], [17]. The near panmixia among ocean-type populations implies high levels of dispersal along shorelines in the NW Pacific and in the NE Pacific. Paradoxically, a sharp genetic boundary around the Alaska Peninsula separates populations in the Bering Sea from the Gulf of Alaska [13]. This boundary likely represents a post-glacial contact zone between populations isolated in southern refugia during late Pleistocene episodes of coastal glaciation [13], but the mechanisms maintaining genetic separating between groups are uncertain.

Surveys of molecular variability among populations have found only small differences between ocean-type populations within the NE and NW Pacific [13]–[15], [18]. Nevertheless, populations with spawning-time differences from other populations [7], populations isolated in fjords [19] and populations adapted to brackish water [14] show moderate genetic differences from open-ocean herring populations. However, these populations are not common. Populations at the margins of the distribution of Pacific herring may also show genetic divergence from central populations because of geographic isolation or adaptation to peripheral environments. In the NE Pacific, the edge of the species distribution has retracted northward since the 1950 s to San Francisco Bay [16]. These populations are genetically similar to more northern populations in the NE Pacific [13], [15]. Less is known about the genetics of herring populations at the southern edge of the distribution in the NW Pacific. Herring appear episodically in the Yellow Sea about every 30 years, because of decadal shifts in temperature and productivity in the Yellow Sea [20]. These environmental shifts lead to short-term cycles of extinction and colonization [20].

The goal of the present study was to use mitochondrial (mt) DNA and microsatellite DNA markers to examine various aspects of population structure in ocean-type populations of Pacific herring across the North Pacific. These two marker types have different mutation rates and enable us to detect contemporary gene flow and deeper separations between populations. Relatively smaller mutation rates for mtDNA allow the reconstructions of population events reaching back several thousand years, and larger mutation rates for microsatellites give insights into contemporary levels of gene flow. One objective was to investigate the population structure of herring in the NW Pacific, and in particular to estimate the relationship of herring in the Yellow Sea to other NW Pacific herring. If present-day populations in the Yellow Sea were established by a small number of founders, these populations may show reduced levels of genetic diversity and departures from neutrality. A second objective was to estimate the population structure of ocean-type herring in the NW Pacific. Previous studies of mtDNA sequence variability [15] and allozymes [13], [14], based on only a few samples, showed no significant differences between ocean-type populations. Here we surveyed variability at nine microsatellite loci, in addition to mtDNA control region sequences in seven samples extending over the range of the NW Pacific lineage of Pacific herring. A third objective was to investigate the contact zone between the NW and NE Pacific lineages of herring. On a large geographic scales, studies using allozyme [13], [14] and mtDNA [15] show a strong genetic break between populations in the Bering Sea and the Gulf of Alaska that appears to reflect a contact zone between refuge populations isolated by Pleistocene coastal glaciations. In contrast to allozymes and mtDNA, microsatellites showed a pattern of isolation by distance, without a strong break across the Alaska Peninsula [18]. However, no NW Pacific samples were included in that study. Here we use mtDNA and microsatellite markers to further investigate this transition zone.

## Materials and Methods

### Ethics Statement

All experimental procedures involving fish were approved by the Institutional Animal Care and Use Committee of Ocean University of China (Permit Number: OUC-20120525). This study did not involve endangered or protected species and the sampling locations are not privately-owned or protected in any way.

### Sample collection

**Figure 1:**
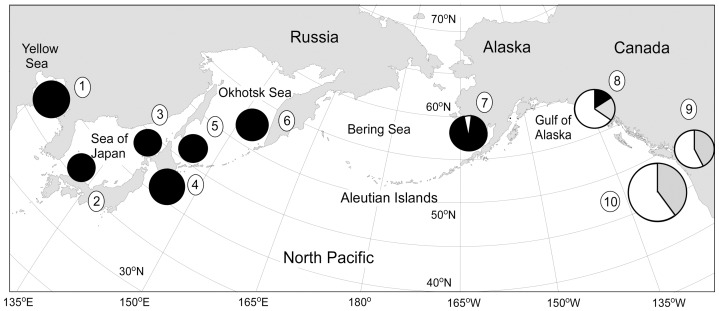
Sampling locations and frequency distributions of the three major haplotype lineages in ten samples. Sample numbers as in Table 1. Key to haplotype lineage: A  =  black; B  =  grey; C  =  white.

Tissue samples from 341 Pacific herring adults were collected in 2003–2010 at six locations in the NW Pacific, one location in the southeastern Bering Sea, and three locations in the NE Pacific (Table 1, Fig. 1). Muscle tissue samples were collected at YS, SH, IB, NH, AK, WK, TO and SI, and fins were collected from fish at SG and CW. A total of 341 fish from ten locations was examined for mtDNA sequence variation, and 214 fish from nine locations were screened for microsatellite DNA variation. Samples from the Strait of Georgia (SG) were excluded from the microsatellite analysis because of poor quality.

**Table 1:** Summary statistics for mitochondrial DNA variability in Pacific herring.

Location	Abbr.	Year	*N*	*n*	*S*		*h*	Θ_π_	π	*F*_S_	*P*
Northwestern Pacific											
1. Rongcheng, Yellow Sea	YS	2003	39	13		24	0.908 ±0.024	0.0095 ±0.0053	4.51 ±2.27	−1.301	0.319
2. Shimane, Sea of Japan	SH	2007	24	18		23	0.975 ±0.019	0.0113 ±0.0063	5.71 ±2.80	−8.427	0.002
3. Ishikari Bay, Sea of Japan	IB	2005	24	12		16	0.935 ±0.025	0.0106 ±0.0059	5.06 ±2.55	−1.635	0.242
4. North of Hokkaido, Okhotsk Sea	NH	2004	26	15		27	0.945 ±0.024	0.0107 ±0.0060	5.11 ±2.56	−4.135	0.041
5. Akkeshi, East of Hokkaido	AK	2005	36	26		38	0.975 ±0.014	0.0120 ±0.0065	5.71 ±2.80	−15.903	<0.001
6. West Kamchatka, Okhotsk Sea	WK	2007	29	19		24	0.948 ±0.028	0.0109 ±0.0061	5.24 ±2.61	−7.992	0.004
7. Togiak, S.E. Bering Sea	TO	2007	30	14		33	0.894 ±0.037	0.0120 ±0.0066	5.76 ±2.84	−1.976	0.208
Northeastern Pacific											
8. Sitka, S.E. Alaska	SI	2007	26	20		52	0.963 ±0.027	0.0223 ±0.0117	10.84 ±5.10	−7.654	0.005
9. Strait of Georgia	SG	2009	24	20		37	0.978 ±0.021	0.0204 ±0.0108	9.90 ±4.69	−24.490	<0.001
10. Coastal Washington	CW	2005	84	61		69	0.980 ±0.008	0.0227 ±0.0116	11.02 ±5.06	−1.301	0.319

Sample localities, sample abbreviation, collection year, sample size (*N*), number of haplotypes (*n*), number of segregating sites (*S*), haplotype diversity (*h*, standard deviation), nucleotide diversity (Θπ, standard deviation; TrN model of mutation), mean number of nucleotide mismatches (π), Tajima's *D*, and Fu's *F*_S._

### DNA amplification

Genomic DNA was extracted following Sambrook et al. [21]. The first hypervariable segment of the mtDNA CR was amplified with the primers CP-F: 5′-ACTCCCAAAGCTAGGATTCTG-3′ (forward) and CP-R: 5′-CTCAATTTGGTTGGTCGGTTC-3′ (reverse), which amplified a 480 base pair (bp) fragment. Polymerase chain reaction (PCR), purification of PCR product, and sequencing were carried out according to protocols in J.-X. Liu et al. [22]. Sequences were edited and aligned using DNASTAR (DNASTAR Inc., www.dnastar.com) and deposited in the GenBank (Accession numbers JN796250-JN796404) (Table S1).

Ten microsatellite loci were amplified with primers developed by Olsen et al. [23] and O'Connell et al. [18] (Table S2) and with the PCR conditions in Olsen et al. [23]. PCR products were separated in a 6% denaturing polyacrylamide gel and visualized with silver staining following Bassam et al. [24]. Alleles for a locus were numbered by relative electrophoretic distance [25].

### MtDNA sequence analysis

Molecular diversity indices were estimated with ARLEQUIN 3.1 [26]. ModelTest 3.7 [27] indicated that the TrN+I+G model [28] nucleotide substitution model best fit the sequences with invariable sites *I* = 0.60 and gamma shape parameter α = 0.66. A 95% confident parsimony haplotype network was constructed with TCS 1.15 [29].

Genetic divergence between populations was estimated with an analysis of molecular variation (AMOVA) [30] using *F*_CT_ and Φ_CT_ in ARLEQUIN. Several models of population structure, based on adjacent-sample pooling, were considered to assess population structure. Genetic breaks between populations were identified by finding the largest significant values of *F*_CT_ or Φ_CT_ between population groups and the smallest and non-significant values of *F*_SC_ and Φ_SC_ among populations within these groups.

Deviations from neutrality were tested with Fu's *F*_S_ [31], as implemented in ARLEQUIN. Nucleotide mismatch distributions (ARLEQUIN) were used to test for population growth and spatial range expansions [32] and to estimate demographic parameters representing population sizes before (θ_0_) and after (θ_1_) population growth, and a compound variable representing the time since population expansion, τ = 2*ut*, where *u* is the mutation rate over the whole sequence and *t* is the time since population growth or geographical expansion [33], [34]. However, since a molecular clock calibration is uncertain, we used the mismatch distributions to compare the general demographic histories among populations.

Isolation by distance was tested by comparing standardized genetic distance values (*F*_CT_ and Φ_CT_) between samples and geographical distance with Mantel's test for correlation between difference matrices in the online program IBDWS (isolation by distance web service at http://ibdws.sdsu.edu/~ibdws/) [35], [36]. The probability that the correlation (*r*) between genetic and geographical distances was larger than 0.0 was estimated with 1000 randomizations. Genetic distances, or geographical distances, or both, were log transformed to attempt to improve correlations. A log transformation of geographical distances tested for IBD on large geographical scales, and a log transformation of genetic distances tested for IBD at large values of genetic distance.

### Microsatellite DNA data analysis

We calculated the number of alleles, observed and expected heterozygosities with POPGENE 1.32 [37]. Fit to Hardy-Weinberg genotypic expectations (HWE) and genotypic linkage disequilibrium were tested with GENEPOP 4.0 [38] with 10 000 burn-in steps, and 500 batches of 5000 Monte Carlo Markov Chain (MCMC) steps per batch. Departures from neutrality of microsatellite loci were tested using LOSITAN. The analysis was made on two datasets. The first included the seven samples from the NW Pacific. The second was made on all nine samples from across the North Pacific. We used individual assignment tests implemented in STRUCTURE 2.1 [39] and followed Evanno et al. [40] to estimate the number of genetically discrete populations. Population groups were simulated for K = 1 to 9, assuming possible mixed ancestry and correlated allele frequencies, and using 1 000 000 MCMC steps, discarding the first 100 000 steps.

We used POWSIM 4.0 [41] to estimate statistical power to detect various levels of differentiation with the 10 microsatellites in Pacific herring. Burn-in consisted of 1000 steps followed by 100 batches of 1000 steps. *X* ^2^ and Fisher's probabilities were used to test the significance of an *F*_CT_ value for each replicate run, and the proportion of significant *F*_CT_ values among 1000 replicate simulations yielded statistical power for a given level of divergence. Divergence among populations was modelled by allowing frequencies to drift for a given number of generations with effective population sizes (*N*_e_) equal to 2000 fish. The results of the simulations indicated a rapid increase in power with larger values of *F*_CT_ (Table 2). Significant *F*_CT_ values of 0.004 or larger were detected with about 90% confidence and significant values larger than *F*_CT_  = 0.008 were detected with 100% confidence.

**Table 2:** Results of simulations to test for statistical power of 10 microsatellites to detect significant genetic population structure with *F*_CT_ and X^2^ and Fisher's probabilities of testing for *F*_CT_ >0.0.

*F*_CT_	X^2^	Fisher
**0.001**	0.199	0.199
**0.002**	0.520	0.518
**0.004**	0.953	0.909
**0.008**	1.0	1.0
**0.016**	1.0	1.0

We used several models of population structure and AMOVA in ARLEQUIN to identify genetic breaks among populations. Pairwise population *F*-statistics were calculated with ARLEQUIN and were used to test for a correlation between standardized values of *F*_CT_/(1-*F*_CT_) [35] and geographical distance between sampling sites to test for isolation by distance [42]. Mantel's test for isolation by distance (IBD) among populations was performed using IBDWS [36]. The probability that a correlation was significantly larger than 0.0 was tested with 1000 randomizations. Log transformations of the variables were made to attempt to improve the correlation.

## Results

### Mitochondrial DNA variation

After a conserved 17 bp portion of the tRNA^Pro^ gene at 5′ end was removed, 463 bp CR sequences were obtained for 342 specimens. These sequences were polymorphic at 110 nucleotide sites (76 parsimony informative sites), with 93 transitions, 28 transversions and six indels, which defined 155 haplotypes (113 singletons). Haplotype diversity ranged from *h* = 0.894 (TO) to 0.980 (CW) (mean *h* = 0.979), and nucleotide diversity (Θ_π_) ranged from 0.0098 (YS) to 0.0238 (CW) (mean Θ_π_  = 0.0208) (Table 1). Nucleotide diversities were larger in the three coastal American samples (SI, SG and CW) south of the Alaska Peninsula than in the seven samples from the Northwestern Pacific and southeastern Bering Sea (Table 1).

**Figure 2:**
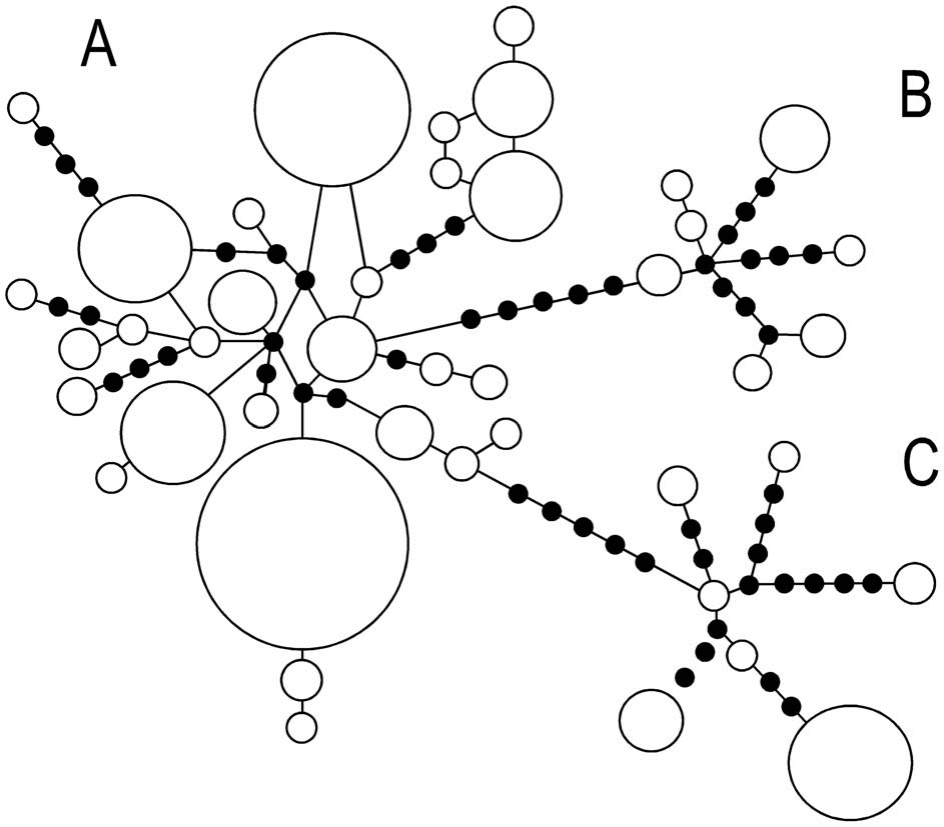
Parsimony-based mitochondrial DNA haplotype networks excluding singleton haplotypes. The sizes of open circles are proportional to haplotype frequency. Closed circles represent hypothetical but unobserved haplotypes.

The larger nucleotide diversities in the NE Pacific were due to the presence of two deeply separated mtDNA lineages. Haplotypes fell into three previously described lineages (Fig. 2). Lineage A occurred almost exclusively in the NW Pacific and Bering Sea, but with a few individuals in the Gulf of Alaska, and lineages B and C co-occurred in the NE Pacific (Fig. 1). Sequence divergences between lineages were 1.6% (S.E. 0.6%) between A and B, 1.9% (0.6%) between A and C, and 1.8% (0.6%) between B and C. Fu's *F*_S_ was highly significant (*P*<0.001) for each lineage, and was significant for all the samples, except for YS, IB, NH, TO, and CW (Table 1).

Values of *F*_CT_ between samples from the NW and NE Pacific varied from 0.023 to 0.064 and most were significant after Bonferroni correction (Table 3). In the NW Pacific, *F*_CT_ varied from 0.007 to 0.059 between samples, and 3 of the 6 comparisons involving YS were significant. None of the remaining comparisons was significant in the NW Pacific, nor in the NE Pacific. All of the pairwise values of Φ_ST_ between NW Pacific and NE Pacific samples were significant (Φ_CT_  = 0.331–0.459), and most of the comparisons involving YS were significant (Φ_CT_  = 0.039–0.108), except between YS and AK. Two comparisons, YS*-*NH and YS*-*TO, were not significant after Bonferroni correction.

**Table 3:** Pairwise *F*_CT_ (below diagonal) and Φ_CT_ (above diagonal) with the TrN model of mutation between samples of Pacific herring.

	YS	SH	IB	NH	AK	WK	TO	SI	SG	CW
**YS**		**0.081**	**0.097**	0.039	0.014	**0.108**	**0.051**	**0.459**	**0.372**	**0.400**
**SH**	0.023		0.004	−0.001	0.010	0.017	0.000	**0.433**	**0.353**	**0.385**
**IB**	**0.046**	0.004		0.025	0.023	0.065	0.011	**0.438**	**0.358**	**0.384**
**NH**	0.017	−0.006	0.013		−0.018	−0.009	−0.015	**0.432**	**0.347**	**0.382**
**AK**	**0.034**	0.005	0.020	−0.007		0.019	−0.004	**0.429**	**0.346**	**0.385**
**WK**	**0.059**	0.003	0.026	0.006	0.007		−0.004	**0.429**	**0.365**	**0.400**
**TO**	0.033	0.017	0.029	−0.003	0.021	0.024		**0.416**	**0.331**	**0.373**
**SI**	**0.058**	**0.024**	**0.043**	**0.039**	**0.024**	**0.037**	**0.064**		0.014	−0.003
**SG**	**0.058**	0.025	**0.042**	0.036	**0.026**	0.037	0.056	−0.008		0.043
**CW**	**0.055**	**0.023**	**0.041**	**0.037**	**0.023**	**0.035**	**0.060**	−0.001	−0.002	

Bold values are significant after Bonferroni adjustment of rejection probability.

An AMOVA of haplotype variability among samples yielded an *F*_CT_  = 0.029 (*P*<0.001) overall, but divergence was much larger, Φ_CT_  = 0.297 (*P*<0.001), when divergence between haplotypes was considered (Table 4). This diversity was first partitioned into two groups, corresponding to the major genetic discontinuity between populations, and this yielded significant, or nearly significant, statistics between the two groups (*F*_CT_  = 0.029, *P* = 0.008; Φ_CT_  = 0.420, *P* = 0.053). The AMOVA for the three NE Pacific samples, detected no significant divergence among three samples extending from Sitka, Alaska to Washington (*F*_CT_  = −0.002, *P* = 0.638; Φ_CT_  = 0.021, *P* = 0.053).

**Table 4:** AMOVAs of models of population structure based on mitochondrial DNA frequencies (*F*_CT_) and TrN distances (α = 0.6) between haplotypes (Φ_CT_) in samples of Pacific herring.

YS	SH	IB	NH	AK	WK	TO	SI	SG	CW	*F*_CT_	*P*	*F*_SC_	*P*	*F*_ST_	*P*	Φ_CT_	*P*	Φ_SC_	*P*	Φ_ST_	*P*
[*	*	*	*	*	*	*	*	*	*]	0.029	<0.001	–	–	–	–	0.297	<0.001	–	–	–	–
[*	*	*	*	*	*	*]	[*	*	*]	0.029	0.0077	0.013	0.0004	0.042	<0.001	0.420	0.053	0.019	0.003	0.431	<0.001
[*	*	*	*	*	*]	[*	*	*	*]	0.012	0.069	0.022	<0.001	0.034	<0.001	0.286	0.012	0.136	<0.001	0.383	<0.001
[*	*	*	*	*	*]	[*]	[*	*	*]	0.026	0.0049	0.012	0.0002	0.038	<0.001	0.375	0.005	0.023	<0.001	0.389	<0.001
–	–	–	–	–	–	–	[*	*	*]	−0.002	0.638	–	–	–	–	0.021	0.053	–	–	–	–
[*	*	*	*	*	*	*]	–	–	–	0.019	0.00014	–	–	–	–	0.027	0.002	–	–	–	–
[*]	[*	*	*	*	*]	[*]	–	–	–	0.020	0.097	0.008	0.089	0.028	0.0001	0.022	0.240	0.014	0.101	0.036	0.002
[*	*	*	*	*	*]	[*]	–	–	–	−0.0005	0.572	0.019	0.0002	0.020	0.0001	−0.022	0.715	0.033	0.001	0.012	0.002
[*	*	*	*	*]	[*	*]	–	–	–	0.008	0.380	0.023	0.009	0.031	0.002	0.002	0.478	0.019	0.0007	0.021	0.0001
[*	*	*	*]	[*	*	*]	–	–	–	0.003	0.315	0.018	0.001	0.021	0.0003	−0.003	0.655	0.029	0.003	0.026	0.002
[*	*	*]	[*	*	*	*]	–	–	–	0.007	0.087	0.016	0.004	0.022	0.0002	0.005	0.340	0.024	0.008	0.029	0.002
[*	*]	[*	*	*	*	*]	–	–	–	0.009	0.095	0.015	0.005	0.024	0.0002	0.006	0.428	0.024	0.010	0.006	0.002
[*]	[*	*	*	*	*	*]	–	–	–	0.024	0.141	0.011	0.023	0.035	0.001	0.048	0.147	0.009	0.168	0.057	0.002

Location abbreviations as in Table 1.

**Figure 3:**
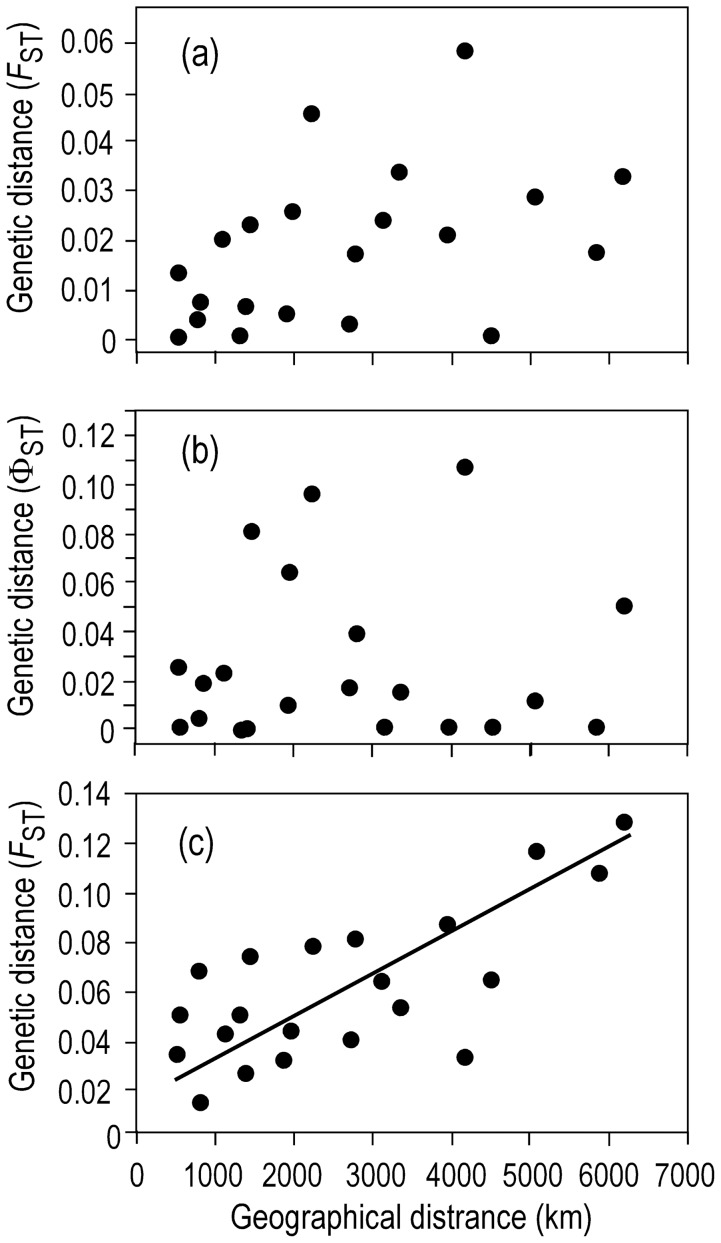
Pairwise estimates of genetic distance and geographical distance in Pacific herring. a) Comparison of pairwise control region mitochondrial DNA *F*_ST_/(1-*F*_ST_) and geographical distance; *r* = 0.428 (*P* = 0.125). b) Comparison of pairwise control region mitochondrial DNA Φ_ST_/(1- Φ_ST_) and geographical distance; *r* = 0.057 (*P* = 0.352). c) Comparison of pairwise *F*_ST_/(1-*F*_ST_) based on nine microsatellite loci and geographic distance; *r* = 0.664 (*P* = 0.001).

In the NW Pacific, significant divergence was detected among the 7 samples extending from the Yellow Sea to the southeastern Bering Sea (*F*_CT_  = 0.019, *P* = 0.0001; Φ_CT_  = 0.027, *P* = 0.002); however, none of the *F*_CT_ and Φ_CT_ values between adjacent groups was significant (Table 4). Nevertheless, most values of *F*_SC_ and Φ_SC_ in these comparisons were significant, indicating fine-scale differences among samples that were not apparent when samples were pooled for regional comparisons. Tests for IBD were not significant for either *F*_CT_/(1-*F*_CT_) (*P* = 0.125) or Φ_CT_/(1-Φ_CT_) (*P* = 0.352) (Fig. 3a, b). Log transformations of genetic distance, geographical distance, or both, did not improve the correlations.

**Figure 4:**
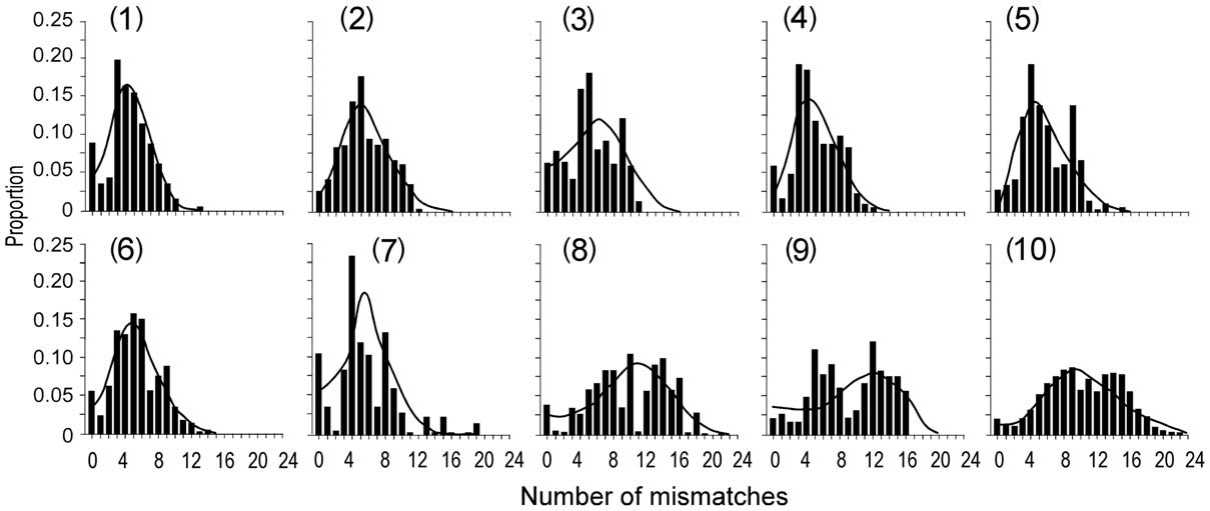
Observed (vertical bars) mtDNA control region mismatch distributions in Pacific herring. Curve represents the expected mismatch distributions under the sudden expansion model. Sample numbers as in Table 1.

**Figure 5:**
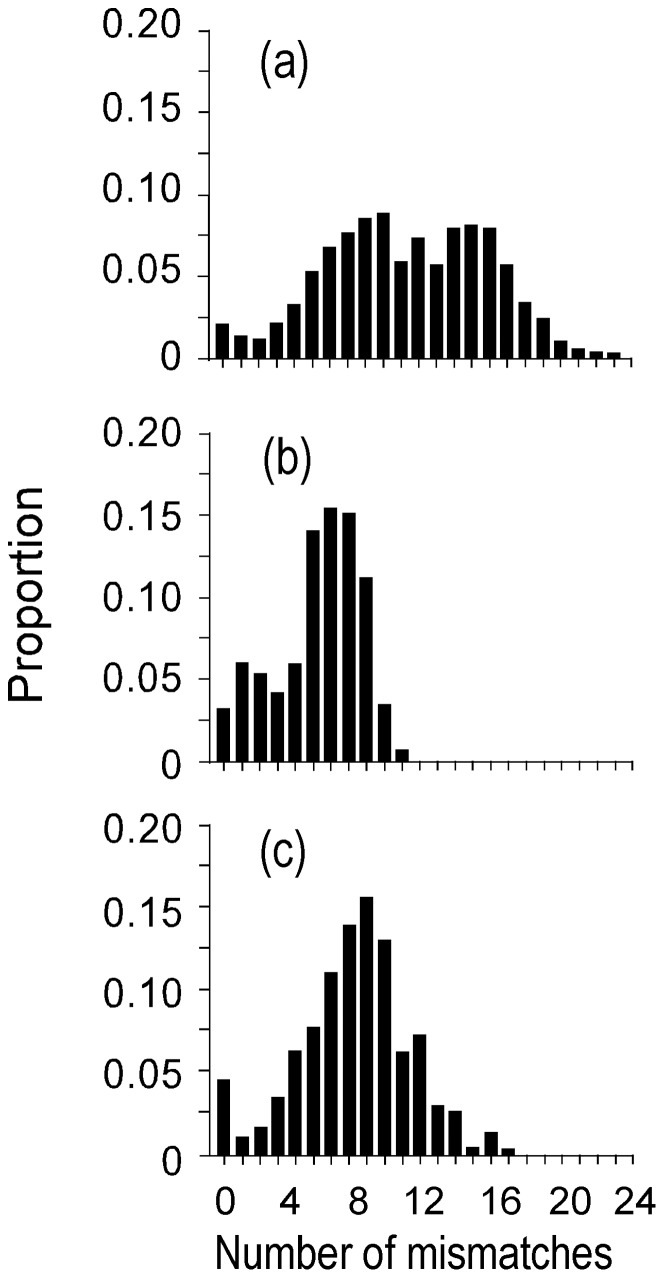
Observed mismatch distributions of control region mtDNA haplotypes in Pacific herring from Coastal Washington. (a) mismatch distribution of haplotypes including lineages B and C. (b) mismatch distribution of haplotypes including lineages B. (c) mismatch distribution of haplotypes including lineages C.

Additional analyses of the mtDNA sequences provided insights in the historical demographies of Pacific herring populations. The shapes of the sequence mismatch distributions were unimodal in YS and SH, but tended to be multimodal in more northern samples (IB, AK, NH, WK, TO) (Fig. 4). However, none of the distributions deviated from an expansion model. The sudden population expansion model could not be distinguished from the spatial population expansion model. The average number of mismatches among samples ranged from 4.43 (YS) to 5.64 (AK) in the NW Pacific, but from 9.29 (SG) to 10.34 (CW) in the NE Pacific (Table 5). However, the samples from the NE Pacific encompassed two haplotype lineages that, when analyzed together, produced larger estimates of demographic parameters than for individual lineages. Only the sample size for CW was large enough to split the sample into separate lineages for additional analysis. The mismatch distributions for lineages B and C in CW were unimodal (Fig. 5); however, the mean number of mismatches was somewhat larger in lineage C (π = 7.30) than in lineage B (π = 5.88). Not all of the tests for neutrality were significant, even though most of the mismatch distributions were unimodal, indicating recent population expansions. *F*_S_ was significant in samples from SH, NH, AK and WK in the NW Pacific, and in samples SI and SG in the NE Pacific.

**Table 5:** Estimates of demographic parameters from control region mtDNA mismatch distributions in samples of Pacific herring.

	Sudden population expansion				Spatial population expansion		
Sample	Mean	Τ	Θ_0_	Θ_1_	Mean	Θ	M
**YS**	4.43	5.020	0.000	15.300	5.780	0.001	11.328
**SH**	5.58	4.290	1.896	58.359	3.974	2.140	53.888
**IB**	5.21	5.266	1.287	17.368	3.993	2.329	11.721
**NH**	5.09	3.613	1.893	92.344	3.644	1.726	23.094
**AK**	5.64	3.654	2.208	9999	3.711	2.110	63.609
**WK**	5.30	4.293	1.454	40.938	4.002	1.584	21.217
**TO**	5.55^a^	4.965	0.954	19.473	4.533	0.971	7.900
**SI**	10.19	10.707	1.000	38.092	8.154	3.838	23.095
**SG**	9.29	13.344	0.014	24.702	5.005	7.224	55.805
**CW**	10.34	7.861	4.479	62.148	6.768	5.511	44.238
**CW-B**	5.88	7.000	0.001	47.812	6.808	0.029	37.361
**CW-C**	7.30	7.982	0.000	62.656	7.771	0.027	20.843

a Significant deviation from sudden population expansion model (*P*<0.05). CW-B represent the individuals belong to lineage B, and CW-C represent the ones belong to lineage C.

### Microsatellite variation

Eight microsatellite loci developed for Pacific herring, and two loci developed for the sister species Atlantic herring, *C. harengus*, were used to estimate divergences between populations. The average number of alleles per locus (*A*) over samples ranged from 2.2 alleles for *Cpa 110* to 13.6 for *Cha 17* (Table S2). Heterozygosities were lower on average among samples from the NW Pacific (*H*_E_  = 0.672–0.807) than among those from the NE Pacific (*H*_E_  = 0.792–0.811). No geographical trend appeared overall in the levels of microsatellite diversity. However, the lowest levels of diversity appeared in the sample from the Yellow Sea. Ten of 100 locus-by-population tests exhibited significant departures from HWE with heterozygote excess, but none remained significant after correction for multiple tests. Locus *Cpa110* was excluded from further analysis because significant linkage disequilibrium appeared between *Cpa110* and *Cpa108*, *Cpa110* and *Cha17* over all populations.

**Figure 6:**
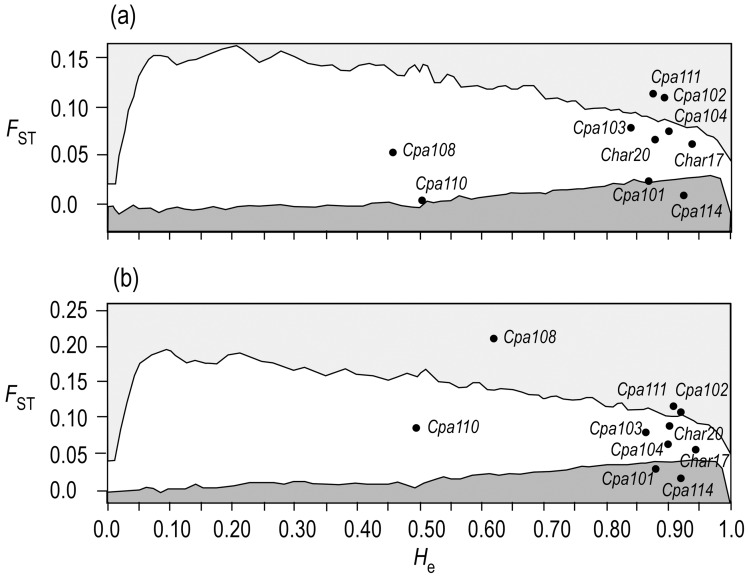
Distribution of 10 microsatellite loci along *F*_ST_ and *H*_e_ axes and 95% confidence interval. (a) LOSITAN analysis of seven samples from the NW Pacific. (b) LOSITAN analysis of nine samples across the North Pacific. The upper and lower shaded areas represent confidence intervals for expected distributions of loci under selection.

The LOSITAN analysis for the seven samples from the NW Pacific detected significant departures for *Cpa111* and *Cpa102* that indicate possible directional selection (Fig. 6a). Divergence was greater than expected for a given level of heterozygosity for these loci. In contrast, *Cpa114* showed significantly less divergence than expected, a condition that may be due to balancing selection. When the analysis was performed on all nine samples from across the North Pacific (Fig. 6b), loci Cpa111 and Cpa102 again showed departures from neutrality, as did locus Cpa108, indicating the potential effect of directional selection. Cpa114 and Cpa101 both exhibited less divergence than expected, and may show the possible results of balancing selection.

**Figure 7:**
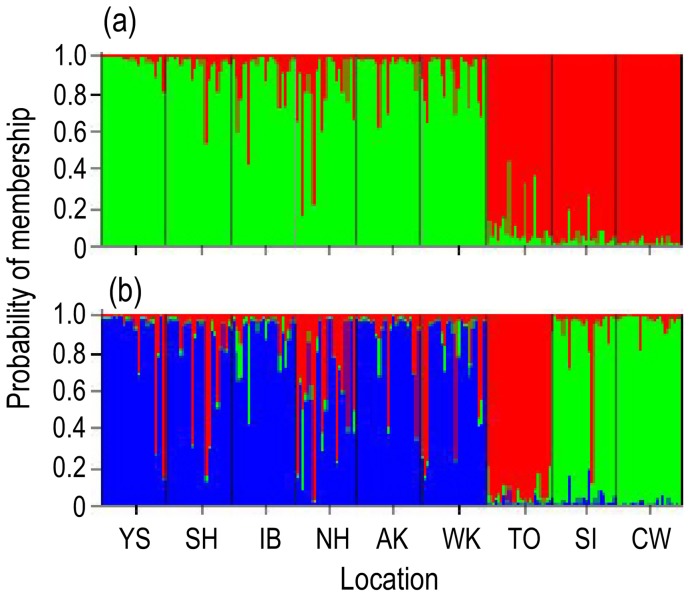
Results of STRUCTURE analysis of 10 microsatellite loci in Pacific herring. Vertical lines are proportional to the probability of individual membership in simulated cluster. (a) *K* = 2, (b) *K* = 3.

STRUCTURE was used to test for population structure assuming one (*K* = 1) to nine (*K* = 9) population groups. The largest Δ*K* appeared under the assumption of two groups. This analysis showed a large genetic discontinuity between NW Pacific and NE Pacific populations, but unexpectedly placed the sample from TO (eastern Bering Sea) into the NE Pacific population group (Fig. 7a). Under the assumption of three groups, the TO sample represented a third group distinct from both the NW and NE Pacific population groups (Fig. 7b).

Tests for divergence between populations, based on the ten microsatellite loci, indicated that all of the pairwise values of *F*_CT_ were significant, except for three comparisons (SH-AK, YS-WK, AK-WK) (Table 6). Divergences (*F*_CT_) ranged from 0.018 to 0.084 between the six samples from the NW Pacific. Divergences between samples from the NW and NE Pacific were generally larger, ranging from 0.070 to 0.168. In the NE Pacific, a small *F*_CT_ value (0.036) was observed between SI and CW (Table 6). An AMOVA of the nine samples yielded an overall *F*_CT_  = 0.073 (*P*<0.001) (Table 7). The partition of samples into two geographic groups, the NW Pacific group (including YS, SH, IB, NH, AK, WK) and the NE Pacific group (including TO, SI, CW), yielded the largest *F*_CT_ (0.057, *P* = 0.011) and the smallest *F*_SC_ (0.045, *P*<0.001). The significant *F*_SC_ value also indicated differences within the two groups. When TO was separated from NE Pacific group, *F*_CT_ was also large and significant (*F*_CT_  = 0.046, *P* = 0.004). This echoes the results of the STRUCTURE analysis for *K* = 3. Less divergence was found between TO and the two samples from NE Pacific group than between TO and the six samples from NW Pacific group (Table 7). A significant correlation between genetic *F*_CT_/(1-*F*_CT_) and geographical distance appeared between samples in the NW Pacific (*r* = 0.625, *P* = 0.043) and across the whole geographical range (*r* = 0.664, *P*<0.001, Fig. 3c).

**Table 6:** Pairwise values of *F*_CT_ based on nine microsatellite loci in Pacific herring.

	YS	SH	IB	NH	AK	WK	TO	SI
SH	**0.078**							
IB	**0.084**	**0.075**						
NH	**0.082**	**0.056**	**0.054**					
AK	**0.052**	0.036	**0.045**	**0.038**				
WK	0.032	**0.045**	**0.047**	**0.030**	0.018			
TO	**0.132**	**0.117**	**0.123**	**0.070**	**0.093**	**0.069**		
SI	**0.143**	**0.112**	**0.095**	**0.077**	**0.089**	**0.077**	**0.078**	
CW	**0.168**	**0.125**	**0.104**	**0.078**	**0.096**	**0.108**	**0.105**	**0.036**

Bold values indicate significance (*P*<0.05) after Bonferroni correction of probabilities.

**Table 7:** AMOVAs of models of population structure based on frequencies at nine microsatellite loci in samples of Pacific herring.

YS	SH	IB	NH	AK	WK	TO	SI	CW	*F*_CT_	*P*	*F*_SC_	*P*	*F*_ST_	*P*
[*	*	*	*	*	*	*	*	*]	0.073	<0.001	–	–	–	–
[*	*	*	*	*	*	*]	[*	*]	0.023	0.113	0.065	<0.001	0.086	<0.001
[*	*	*	*	*	*]	[*	*	*]	0.057	0.012	0.045	<0.001	0.100	<0.001
[*	*	*	*	*	*]	[*]	[*	*]	0.046	0.004	0.048	<0.001	0.092	<0.001
–	–	–	–	–	–	[*]	[*	*]	0.007	0.332	0.018	0.006	0.025	<0.001
[*	*	*	*	*	*]	[*]	–	–	0.063	0.142	0.053	<0.001	0.113	<0.001
[*	*	*	*	*	*]	–	–	–	0.053	<0.001	–	–	–	–
[*	*	*	*	*]	[*]	–	–	–	−0.021	1.000	0.050	<0.001	0.040	<0.001
[*	*	*	*]	[*	*]	–	–	–	−0.008	0.735	0.053	<0.001	0.050	<0.001
[*	*	*]	[*	*	*]	–	–	–	−0.012	0.900	0.060	<0.001	0.049	<0.001
[*	*]	[*	*	*	*]	–	–	–	−0.001	0.535	0.057	<0.001	0.053	<0.001
[*]	[*	*	*	*	*]	–	–	–	0.011	0.332	0.060	<0.001	0.060	<0.001

Location abbreviations as in Table 1.

## Discussion

The results of this study provide insights into two general features of Pacific herring population structure. First, our estimates of population structure in the western North Pacific with control region mtDNA sequences and microsatellites show minimal population structure, except for the divergence of Yellow Sea populations from other NW Pacific herring populations. Second, our results for mtDNA confirm the presence of a genetic discontinuity between the eastern Bering Sea and Gulf of Alaska populations of Pacific herring. However, microsatellite markers show a discordant biogeographic pattern from mtDNA markers, in that Eastern Bering Sea herring are more similar to populations in the Gulf of Alaska than to NW Pacific populations.

Before discussing these findings in detail, we mention two caveats. First, even though sample sizes for microsatellites were small, these samples sizes were still large enough and the microsatellite marker polymorphic enough to provide power to detect population structure as low as *F*_CT_  = 0.004 about 90% of the time. This level of divergence is much small that the level of divergence between the NW Pacific and NE Pacific genetic groups, and hence our results can provide further insights into the nature of the genetic discontinuity across the North Pacific. A second caveat is that, although we sampled ocean-type herring at several locations in the NW Pacific, we had fewer samples from the NE Pacific. Hence, our focus is on estimating population structure in the NW Pacific. Nevertheless, our array of samples allows us to further examine the genetic discontinuity across the N Pacific.

### Northwestern Pacific herring

Several oceanic and biological traits potentially influence contemporary levels of population structure. Shoreline complexity on regional scales may isolate populations. Semi-enclosed Okhotsk and Japan seas, numerous island archipelagos and peninsulas, such as the Korean Peninsula, potentially act as barriers to dispersal between populations. The attachment of early embryonic stages to kelps and sessile invertebrates can also reduce dispersal [43]. After hatching, larval dispersal is influenced by local current patterns, which can be dispersive or retentive, depending on inshore current systems. Juveniles usually move offshore after their first summer but recruit later into the adult population [44]. Given these restrictions on dispersal, we would expect to find at least some genetic differences between ocean-type herring populations. However, our results detected only small amounts of population structure among populations of ocean-type herring in the western North Pacific.

In our study, the overall AMOVAs for both mtDNA (*F*_CT_  = 0.019, *P* = 0.0001; Φ_CT_  = 0.027, *P* = 0.002) and microsatellites (*F*_CT_  = 0.053, *P*<0.001) showed significant differences among populations in the NW Pacific, but particular comparisons between population groups did not show a clear geographical patterns. Nevertheless, isolation by distance (IBD) was detected for microsatellites (*r* = 0.625, *P* = 0.04), but was absent for mtDNA *F*_CT_ (*r* = 0.428, *P* = 0.125) and Φ_CT_ (*r* = 0.057, *P* = 0.351). This contrast may be due to differences in the mutation rates between the two markers. The larger mutation rates in microsatellites may allow the detection of short-term isolations between populations. For example, the seasonal movement of herring from offshore overwintering areas to inshore spawning areas [44] tends to isolate populations to some extent on short time scales, and this isolation is sufficient to lead to IBD for microsatellites. The geographical scale of IBD is expected to be large, because geographical scales of seasonal migration pathways can be on the order of hundreds of kilometers [45].

Genetic differences among populations appear to be due in part to isolation spawning-time differences among populations and because of adaptive responses to local habitat conditions [7], [17], [19]. Geographic differences in morphometric and meristic variables [44], [46], [47] and in otolith elemental compositions [48] among populations support the hypothesis of adaptive responses to local environments and short-term isolation between populations. On longer time scales, shifts in annual migration patterns in response to ocean-climate variability leads to mixing between herring aggregations [45], [49], [50]. This mixing tends to homogenize the frequencies of markers, such as mtDNA, with relatively smaller mutation rates [11].

The sample from the Yellow Sea was drawn from Pacific herring living at the edge of the species' distribution. These fish had marginally lower genetic diversities for both mtDNA (Θ_π_  = 0.0095 vs Θ_π_  = 0.0107–0.0120) and microsatellite (*H*_E_  = 0.672 vs *H*_E_  = 0.736–0.811). No significant mtDNA differentiations were detected among different samples in previous study [51] indicating the populations in the Yellow Sea were likely a single panmictic stock. The lower genetic diversity may reflect population bottlenecks from environmental variability at the edge of the species' distribution [52]. Population oscillations can lead to the loss of genetic variability, depending on the level of dispersal from central populations [53]. Pacific herring appear episodically in the Yellow Sea about every 30 years [20], [51] and the low level of differentiation from other NW Pacific populations may be due to a recent colonization of the Yellow Sea.

### Historical demography

Pleistocene glaciations produced coastal glaciers and lower sea levels [54], [55] that would have had a profound influence on the distributions and abundances of Pacific herring populations. Most of our samples were taken from areas that only recently have been available for the shallow-water populations of herring, and, hence, these populations have colonized northern areas since the last glacial maximum about 18 thousand years ago. The mismatch distribution for each of the samples shows a unimodal distribution indicating recent population growth, but the analyses could not distinguish between the geographic expansion of a stable population or population growth. Given the historical setting, an expansion of a refuge population into newly opened northern habitats is the most likely interpretation of the unimodal mismatch distributions.

The lack of an accurate mutation rate prevents us from making a molecular-clock calibration to time the population expansion. However, the uniformity in the mean number of mismatches among NW Pacific populations (π = 4.51–5.76) indicates that post-glacial population expansions occurred at about the same time. In the NE Pacific, however, mismatches for the lineages B and C were larger than those for NW Pacific populations (π = 9.90–11.02), suggesting either an earlier expansion, or differences in metapopulation structure between the two regions. The NW Pacific experiences greater annual and decadal swings in climate and temperature than does the NE Pacific [56]–[58]. Both smaller allozyme and microsatellite diversity may results from a greater incidence of local extinction and colonization in the NW Pacific. Tests for neutrality with *F*_S_ generally indicated excesses of low-frequency haplotypes that are also consistent with recent population expansions.

### Phylogeographic break across secondary contact zone

Three deep mtDNA lineages occur in Pacific herring that show control-region sequence divergences of 1.5% to 1.9% from one another [15], [16]. Divergences between A in the NW Pacific, and B and C in the NE Pacific likely arose during geographical isolations during Pleistocene glaciations of two groups across the North Pacific [15], [16]. However, the origins of the co-distributed lineages B and C are controversial [15], [16], but are likely due to lineage sorting in a large population and not due divergence in isolation [16]. Lineage A is largely limited to the NW Pacific, but a few fish bearing A-lineage haplotypes have been found in the Gulf of Alaska ([15], this study). Lineages B and C co-occur in the NE Pacific, but also appear in small numbers in the eastern Bering Sea. These ‘stray’ haplotypes likely represent long-distance dispersals.

Previous studies of allozyme markers showed a strong genetic discontinuity between NW and NE Pacific populations [13], [14] that is mirrored by mtDNA markers [15], [16]. Microsatellite markers, on the other hand, tend to show small amounts of contemporary population structure [7], [17]–[19]. The dynamics of the suture zone between the NW (phylogroup A) and NE (phylogroups A and B) Pacific groups are not well known, in part, because of the difficulty in sampling remote rock shores along the Alaska Peninsula that are exposed to the open ocean. Pacific herring have been sampled off Kodiak Island in two studies [13], [18] and in the northern part of Bristol Bay in the eastern Bering Sea ([7], [13], [15], this study). Allozyme and mtDNA markers show an abrupt haplotype frequency shift between Kodiak Island and Bristol Bay. In the allozyme study, a small sample (*n* = 21) from the southern shore of the Alaska Peninsula showed intermediate between the two regional groups [13]. While this sample was small, 11 polymorphic allozyme loci did not show heterozygote deficits that would indicate Wahlund's effect and the lack of interbreeding between fish from the two groups.

The geographic pattern across the North Pacific for microsatellites in the study of O'Connell et al. [18] and our study differ from that for allozymes and mtDNA. The results of our study shows that the sample from the eastern Bering Sea was more similar to Pacific herring in the Gulf of Alaska than to NW Pacific herring. The results of O'Connell et al. [18] also showed a close relationship between Bering Sea and NE Pacific populations of herring. They found a pattern of isolation by distance among seven samples from the Bering Sea and Gulf of Alaska, but without a genetic discontinuity along shores of the Alaska Peninsula. However, in that study, samples from the NW Pacific were not included, so the amount of differentiation from Asian Pacific herring could not be estimated.

Molecular genetic differences between the two large groups coincide with morphological and ecological differences between the two groups. Hay et al. [20] summarized the results for age-specific growth, recruitment, and population diversity for Pacific herring across the North Pacific and found differences between populations across the North Pacific. NE Pacific herring (Gulf of Alaska to California) are generally smaller, grow more slowly, and reach lower asymptotic weights than NW Pacific herring. In the NE Pacific, size-at-age differs to a small extent with latitude, but this difference is slight compared to the large west-east difference. The sizes of Pacific herring in the NW Pacific are substantially larger than the sizes of NE Pacific herring at all ages. However, Pacific herring in the eastern Bering Sea are intermediate between NW and NE Pacific herring.

The reason for the differences in growth and asymptotic sizes between populations in the NW and NE Pacific are uncertain. Hay et al. [20] postulated that these differences were adaptive and had evolved in response to ecosystem differences in local prey that influence trophic ecology, to the intensity of competition for food, and to different climatic regimes on either side of the Pacific [56], [58]. If so, then it is enigmatic that the biological differences between the western and eastern Pacific far exceed within-region, north-south differences (at least in the NE Pacific where data are available) in populations that experience as much environmental variability as western and eastern populations [20]. If these biological differences are genetically based, it may be misguided to search for associations of biological differences in contemporary habitats, as the regional differences likely evolved in isolation under different ocean regimes during the Pleistocene ice ages.

The strong mtDNA divergence between NW and NE Pacific populations, together with biparentally inherited microsatellites provide clear dispersal markers. The appearance of fish bearing mtDNA lineage A in the NE Pacific and fish with lineages B or C in the NW Pacific can be taken as evidence for long-distance dispersal across the secondary contact zone. However, the temporal scale of this dispersal is difficult to gauge, but dispersal likely occurs in a stepping-stone fashion over generations. Both males and females carry maternal mtDNA, but only females pass their mtDNA on to offspring, so mtDNA lineages in dispersing males die out in one generation. Dispersing females, however, can pass on out-of-basin haplotypes to their offspring. Future analysis of northern populations by sex may give insights into the frequency of long-distance dispersals and may provide insights into the reproductive successes of migrants.

Several species show abrupt genetic discontinuities between populations [3], [8]–[10], [59] that have variously been attributed to physical barriers to dispersal, habitat discontinuity, weak dispersal ability, selection and secondary contact. While secondary contact between previously isolated populations is a likely explanation for the origins of the genetic discontinuity in Pacific herring [13], [15], [16], additional factors must be at play in maintaining the genetic discontinuity between groups. The evolutionarily rapid post-glacial expansion of herring populations, low levels of differentiation among populations and the observation of displaced mtDNA haplotypes indicate that long-distance dispersals can be a feature of Pacific herring biology. It is puzzling then why a sharp genetic boundary still exists between NW and NE Pacific herring.

Several mechanisms have been postulated to account for abrupt genetic discontinuities along more or less continuous marine habitats. One is that established populations resist the immigration of individuals from other populations through competitive exclusion based on adaptive differences between populations [12], such as size, which may reflect other adaptive differences between these two groups [20]. It is uncertain, however, whether the intermediate sizes of Bering Sea herring reflect mixing and hybridization between the two regional groups, or whether the Bering Sea environment itself selects for intermediate-sized herring. On the one hand, the distributions of mtDNA lineages indicate a lack of mixing, but the intermediate position of Bering Sea herring between NW and NE Pacific herring depicted with microsatellites supports the mixing and hybridization hypothesis.

Discordance between mtDNA and nuclear markers in a secondary contact zone is a common feature in many species, and may be due to combinations of sex-biased dispersal asymmetries, incomplete mtDNA lineage sorting, adaptive introgression, demographic disparities between population groups [11], or gamete incompatibility. In Pacific herring, sex-biased dispersal or mating may be facilitated by size differences between residents and immigrants into the contact zone or by directional genetic incompatibilities between the groups. However, it is difficult to devise a model that would explain a microsatellite hybrid zone that is larger than the mtDNA contact zone in Pacific herring, as mtDNA haplotypes are more geographically restricted in the contact zone than are microsatellite markers. Incomplete mtDNA lineage sorting in the previously isolated regional groups is an unlikely explanation because haplotypes from the major phylogroups are not geographically widespread [11]. Another possibility is a large difference in population sizes of the two groups in the contact zone. If adaptive differences between NW and NE Pacific herring are strong, periodic shifts in ocean climate conditions in the central North Pacific [60] may produce a shifting dynamic in relative population abundances of the two groups within the contact zone that leads to different frequency shifts in the markers [11]. Lastly, divergence between gamete surface proteins may play a role in reproductive incompatibilities, as in some other marine organisms [61]–[63].

In conclusion, Pacific herring are characterized by two parapatric genetically distinct groups that are in secondary contact after divergence in isolation during glacial maxima. Allozyme and mtDNA markers generally show genetic homogeneity among ocean-type populations within regions that has been interpreted to indicate significant amounts of gene flow between populations [13]–[15]. The occurrence of out-of-basin mtDNA markers is consistent with long distance dispersals. Microsatellite markers, on the other hand, tend to show more structure that reflects isolation in fjords and adaptive differences between populations. These contemporary restrictions on gene flow typically produce a broad signal of isolation by distance in microsatellites that still indicates substantial gene flow between local populations, perhaps due in part to decadal shifts in population abundances and distributions. These results together contrast with the sharp genetic discontinuity across the Bering Sea and Gulf of Alaska and point to competitive exclusion, mating incompatibilities, or hybrid inferiority as mechanisms maintaining the discontinuity between the two major groups of Pacific herring.

## Supporting Information

Table S1 **Haplotypes distribution of the three lineages in the ten populations.**
(DOCX)

Table S2 **Levels of genetic variability at ten microsatellite loci in the nine populations of Pacific herring and for all populations pooled.**
(DOCX)
