# Supplementary material for: What Maintains the Central North Pacific Genetic Discontinuity in Pacific Herring?
Source: PLoS One. 2012 Dec 28;7(12):e50340. doi: 10.1371/journal.pone.0050340 (PMC3532504; doi:10.1371/journal.pone.0050340)
Supplement: Table S1 — Haplotypes distribution of the three lineages in the ten populations. (DOCX) [file pone.0050340.s001.docx]

Table S1 Haplotypes distribution of the three lineages in the ten populations.

| **Lineage** | Haplotype | Sample | | | | | | | | | |
| --- | --- | --- | --- | --- | --- | --- | --- | --- | --- | --- | --- |
|  |  | YS | SH | IB | NH | AK | WK | TO | SI | SG | CW |
| **A** | 250 | 2 |  |  |  |  |  |  |  |  |  |
|  | 251 | 4 |  |  | 4 | 4 | 1 | 1 |  |  |  |
|  | 252 | 8 | 2 | 3 | 4 | 2 |  | 8 | 1 |  |  |
|  | 253 | 3 |  |  |  |  |  |  |  |  |  |
|  | 254 | 2 |  |  |  |  |  |  |  |  |  |
|  | 255 | 3 |  |  |  |  |  |  |  |  |  |
|  | 256 | 7 | 1 |  |  |  |  |  |  |  |  |
|  | 257 | 3 | 2 |  | 2 | 1 | 3 | 4 |  |  |  |
|  | 258 | 2 |  |  |  |  |  |  |  |  |  |
|  | 259 | 1 |  |  | 1 | 1 |  |  |  |  |  |
|  | 260 | 2 | 3 | 4 | 2 |  | 1 |  |  |  |  |
|  | 261 | 1 |  |  |  |  | 1 |  |  |  |  |
|  | 262 | 1 |  |  |  |  |  |  |  |  |  |
|  | 263 |  | 2 |  | 2 | 1 | 1 |  |  |  |  |
|  | 264 |  | 1 |  |  |  |  |  |  |  |  |
|  | 265 |  | 1 |  |  |  |  |  |  |  |  |
|  | 266 |  | 1 |  |  | 1 |  |  |  |  |  |
|  | 267 |  | 1 |  |  |  |  |  |  |  |  |
|  | 268 |  | 1 |  | 1 | 3 | 2 | 2 |  |  |  |
|  | 269 |  | 1 |  |  |  |  |  |  |  |  |
|  | 270 |  | 1 |  |  |  |  |  |  |  |  |
|  | 271 |  | 2 | 3 | 3 | 3 | 6 | 5 | 1 |  |  |
|  | 272 |  | 1 |  |  |  |  |  |  |  |  |
|  | 273 |  | 1 |  |  |  |  |  |  |  |  |
|  | 274 |  | 1 |  |  |  |  |  |  |  |  |
|  | 275 |  | 1 |  |  |  |  |  |  |  |  |
|  | 276 |  | 1 |  |  |  |  |  |  |  |  |
|  | 277 |  |  | 2 |  |  |  |  |  |  |  |
|  | 278 |  |  | 1 | 1 | 3 |  | 1 |  |  |  |
|  | 279 |  |  | 2 |  |  |  |  |  |  |  |
|  | 280 |  |  | 3 |  | 1 |  |  |  |  |  |
|  | 281 |  |  | 2 |  |  |  |  |  |  |  |
|  | 282 |  |  | 1 |  |  |  |  |  |  |  |
|  | 283 |  |  | 1 |  |  |  | 1 |  |  |  |
|  | 284 |  |  | 1 |  | 1 | 1 | 1 |  |  |  |
|  | 285 |  |  | 1 |  |  |  |  |  |  |  |
|  | 286 |  |  |  | 1 |  | 1 | 1 |  |  |  |
|  | 287 |  |  |  | 1 |  |  |  |  |  |  |
|  | 288 |  |  |  | 1 |  |  |  |  |  |  |
|  | 289 |  |  |  | 1 |  |  | 2 |  |  |  |
|  | 290 |  |  |  | 1 |  |  |  |  |  |  |
|  | 291 |  |  |  | 1 |  | 1 |  |  |  |  |
|  | 292 |  |  |  |  | 1 |  |  |  |  |  |
|  | 293 |  |  |  |  | 1 |  |  |  |  |  |
|  | 294 |  |  |  |  | 1 |  |  |  |  |  |
|  | 295 |  |  |  |  | 1 |  |  |  |  |  |
|  | 296 |  |  |  |  | 1 |  |  |  |  |  |
|  | 297 |  |  |  |  | 1 |  |  |  |  |  |
|  | 298 |  |  |  |  | 1 |  |  |  |  |  |
|  | 299 |  |  |  |  | 1 |  |  |  |  |  |
|  | 300 |  |  |  |  | 1 |  |  |  |  |  |
|  | 301 |  |  |  |  | 1 |  |  |  |  |  |
|  | 302 |  |  |  |  | 1 |  |  |  |  |  |
|  | 303 |  |  |  |  | 1 |  |  |  |  |  |
|  | 304 |  |  |  |  | 1 |  |  |  |  |  |
|  | 305 |  |  |  |  | 1 |  |  |  |  |  |
|  | 306 |  |  |  |  | 1 |  |  |  |  |  |
|  | 307 |  |  |  |  |  | 1 |  |  |  |  |
|  | 308 |  |  |  |  |  | 2 |  |  |  |  |
|  | 309 |  |  |  |  |  | 1 |  |  |  |  |
|  | 310 |  |  |  |  |  | 1 |  |  |  |  |
|  | 311 |  |  |  |  |  | 2 |  |  |  |  |
|  | 312 |  |  |  |  |  | 1 |  |  |  |  |
|  | 313 |  |  |  |  |  | 1 |  |  |  |  |
|  | 314 |  |  |  |  |  | 1 |  |  |  |  |
|  | 315 |  |  |  |  |  | 1 |  |  |  |  |
|  | 316 |  |  |  |  |  |  | 1 |  |  |  |
|  | 317 |  |  |  |  |  |  | 1 |  |  |  |
|  | 318 |  |  |  |  |  |  | 1 |  |  |  |
|  | 335 |  |  |  |  |  |  |  | 1 |  |  |
|  | 336 |  |  |  |  |  |  |  | 1 |  |  |
|  | 337 |  |  |  |  |  |  |  | 1 |  |  |
| **B** | 332 |  |  |  |  |  |  |  | 1 |  | 2 |
|  | 333 |  |  |  |  |  |  |  | 1 |  | 1 |
|  | 334 |  |  |  |  |  |  |  | 2 | 1 | 5 |
|  | 338 |  |  |  |  |  |  |  |  | 1 |  |
|  | 343 |  |  |  |  |  |  |  |  | 1 |  |
|  | 344 |  |  |  |  |  |  |  |  | 1 |  |
|  | 346 |  |  |  |  |  |  |  |  | 1 | 1 |
|  | 347 |  |  |  |  |  |  |  |  | 1 |  |
|  | 349 |  |  |  |  |  |  |  |  | 1 | 3 |
|  | 350 |  |  |  |  |  |  |  |  | 1 |  |
|  | 353 |  |  |  |  |  |  |  |  | 1 | 1 |
|  | 354 |  |  |  |  |  |  |  |  | 1 |  |
|  | 384 |  |  |  |  |  |  |  |  |  | 4 |
|  | 385 |  |  |  |  |  |  |  |  |  | 1 |
|  | 386 |  |  |  |  |  |  |  |  |  | 1 |
|  | 387 |  |  |  |  |  |  |  |  |  | 1 |
|  | 388 |  |  |  |  |  |  |  |  |  | 1 |
|  | 389 |  |  |  |  |  |  |  |  |  | 1 |
|  | 390 |  |  |  |  |  |  |  |  |  | 1 |
|  | 391 |  |  |  |  |  |  |  |  |  | 1 |
|  | 392 |  |  |  |  |  |  |  |  |  | 1 |
|  | 393 |  |  |  |  |  |  |  |  |  | 1 |
|  | 394 |  |  |  |  |  |  |  |  |  | 1 |
|  | 395 |  |  |  |  |  |  |  |  |  | 1 |
|  | 396 |  |  |  |  |  |  |  |  |  | 1 |
|  | 397 |  |  |  |  |  |  |  |  |  | 1 |
|  | 398 |  |  |  |  |  |  |  |  |  | 1 |
|  | 399 |  |  |  |  |  |  |  |  |  | 1 |
|  | 400 |  |  |  |  |  |  |  |  |  | 1 |
|  | 401 |  |  |  |  |  |  |  |  |  | 1 |
|  | 402 |  |  |  |  |  |  |  |  |  | 1 |
|  | 403 |  |  |  |  |  |  |  |  |  | 1 |
|  | 404 |  |  |  |  |  |  |  |  |  | 1 |
| **C** | 319 |  |  |  |  |  |  | 1 |  |  | 1 |
|  | 320 |  |  |  |  |  |  |  | 1 |  |  |
|  | 321 |  |  |  |  |  |  |  | 5 | 3 | 10 |
|  | 322 |  |  |  |  |  |  |  | 1 |  | 2 |
|  | 323 |  |  |  |  |  |  |  | 1 |  |  |
|  | 324 |  |  |  |  |  |  |  | 1 |  |  |
|  | 325 |  |  |  |  |  |  |  | 1 |  |  |
|  | 326 |  |  |  |  |  |  |  | 2 |  |  |
|  | 327 |  |  |  |  |  |  |  | 1 |  |  |
|  | 328 |  |  |  |  |  |  |  | 1 |  |  |
|  | 329 |  |  |  |  |  |  |  | 1 |  |  |
|  | 330 |  |  |  |  |  |  |  | 1 |  |  |
|  | 331 |  |  |  |  |  |  |  | 1 |  |  |
|  | 339 |  |  |  |  |  |  |  |  | 1 |  |
|  | 340 |  |  |  |  |  |  |  |  | 1 |  |
|  | 341 |  |  |  |  |  |  |  |  | 1 |  |
|  | 342 |  |  |  |  |  |  |  |  | 1 | 1 |
|  | 345 |  |  |  |  |  |  |  |  | 1 |  |
|  | 348 |  |  |  |  |  |  |  |  | 1 |  |
|  | 351 |  |  |  |  |  |  |  |  | 1 |  |
|  | 352 |  |  |  |  |  |  |  |  | 1 | 2 |
|  | 355 |  |  |  |  |  |  |  |  |  | 1 |
|  | 356 |  |  |  |  |  |  |  |  |  | 1 |
|  | 357 |  |  |  |  |  |  |  |  |  | 1 |
|  | 358 |  |  |  |  |  |  |  |  |  | 1 |
|  | 359 |  |  |  |  |  |  |  |  |  | 1 |
|  | 360 |  |  |  |  |  |  |  |  |  | 1 |
|  | 361 |  |  |  |  |  |  |  |  |  | 1 |
|  | 362 |  |  |  |  |  |  |  |  |  | 1 |
|  | 363 |  |  |  |  |  |  |  |  |  | 1 |
|  | 364 |  |  |  |  |  |  |  |  |  | 1 |
|  | 365 |  |  |  |  |  |  |  |  |  | 1 |
|  | 366 |  |  |  |  |  |  |  |  |  | 1 |
|  | 367 |  |  |  |  |  |  |  |  |  | 1 |
|  | 368 |  |  |  |  |  |  |  |  |  | 3 |
|  | 369 |  |  |  |  |  |  |  |  |  | 1 |
|  | 370 |  |  |  |  |  |  |  |  |  | 1 |
|  | 371 |  |  |  |  |  |  |  |  |  | 1 |
|  | 372 |  |  |  |  |  |  |  |  |  | 1 |
|  | 373 |  |  |  |  |  |  |  |  |  | 1 |
|  | 374 |  |  |  |  |  |  |  |  |  | 1 |
|  | 375 |  |  |  |  |  |  |  |  |  | 1 |
|  | 376 |  |  |  |  |  |  |  |  |  | 1 |
|  | 377 |  |  |  |  |  |  |  |  |  | 1 |
|  | 378 |  |  |  |  |  |  |  |  |  | 1 |
|  | 379 |  |  |  |  |  |  |  |  |  | 1 |
|  | 380 |  |  |  |  |  |  |  |  |  | 1 |
|  | 381 |  |  |  |  |  |  |  |  |  | 1 |
|  | 382 |  |  |  |  |  |  |  |  |  | 1 |
|  | 383 |  |  |  |  |  |  |  |  |  | 1 |

Haplotype numbers correspond to the last three digits of GenBank accession numbers JN796250–JN796404.
