# Supplementary material for: What Maintains the Central North Pacific Genetic Discontinuity in Pacific Herring?
Source: PLoS One. 2012 Dec 28;7(12):e50340. doi: 10.1371/journal.pone.0050340 (PMC3532504; doi:10.1371/journal.pone.0050340)
Supplement: Table S2 — Levels of genetic variability at ten microsatellite loci in the nine populations of Pacific herring and for all populations pooled. (DOCX) [file pone.0050340.s002.docx]

Table S2 Levels of genetic variability at ten microsatellite loci in the nine populations of Pacific herring and for all populations pooled.

| Locus | Sample | YS | SN | IB | NH | AS | WK | TG | SI | CW |
| --- | --- | --- | --- | --- | --- | --- | --- | --- | --- | --- |
|  | *n* | 24 | 24 | 24 | 23 | 24 | 24 | 24 | 24 | 23 |
| Cpa 101 | A | 7 | 8 | 12 | 9 | 9 | 12 | 11 | 11 | 10 |
|  | *H*E | 0.835 | 0.834 | 0.850 | 0.835 | 0.881 | 0.850 | 0.837 | 0.890 | 0.857 |
|  | *H*O | 0.958 | 0.783 | 0.826 | 0.909 | 0.833 | 0.727 | 0.700 | 0.842 | 0.773 |
| Cpa 102 | A | 9 | 11 | 8 | 9 | 14 | 14 | 14 | 13 | 17 |
|  | *H*E | 0.566 | 0.785 | 0.804 | 0.839 | 0.882 | 0.850 | 0.843 | 0.907 | 0.894 |
|  | *H*O | 0.478 | 0.810 | 0.565 | 0.739 | 0.833 | 0.750 | 0.565 | 0.476 | 0.455 |
| Cpa 103 | A | 5 | 7 | 7 | 8 | 8 | 9 | 7 | 11 | 12 |
|  | *H*E | 0.653 | 0.818 | 0.759 | 0.826 | 0.753 | 0.834 | 0.768 | 0.824 | 0.897 |
|  | *H*O | 0.667 | 0.667 | 0.625 | 0.909 | 0.870 | 0.667 | 0.833 | 0.739 | 0.850 |
| Cpa 104 | A | 8 | 11 | 10 | 10 | 12 | 11 | 11 | 12 | 16 |
|  | *H*E | 0.824 | 0.840 | 0.849 | 0.865 | 0.855 | 0.840 | 0.740 | 0.832 | 0.921 |
|  | *H*O | 0.833 | 0.667 | 0.750 | 0.636 | 0.708 | 0.750 | 0.625 | 0.714 | 0.762 |
| Cpa 108 | A | 4 | 5 | 8 | 6 | 6 | 4 | 6 | 4 | 7 |
|  | *H*E | 0.160 | 0.243 | 0.469 | 0.669 | 0.634 | 0.327 | 0.504 | 0.645 | 0.733 |
|  | *H*O | 0.167 | 0.217 | 0.409 | 0.476 | 0.625 | 0.318 | 0.458 | 0.500 | 0.579 |
| Cpa 110 | A | 2 | 2 | 2 | 3 | 3 | 2 | 2 | 2 | 2 |
|  | *H*E | 0.439 | 0.503 | 0.488 | 0.532 | 0.529 | 0.510 | 0.511 | 0.496 | 0.059 |
|  | *H*O | 0.375 | 0.375 | 0.375 | 0.652 | 0.458 | 0.348 | 0.652 | 0.500 | 0.059 |
| Cpa 111 | A | 8 | 9 | 15 | 12 | 11 | 10 | 6 | 13 | 13 |
|  | *H*E | 0.723 | 0.700 | 0.908 | 0.879 | 0.860 | 0.877 | 0.477 | 0.902 | 0.895 |
|  | *H*O | 0.667 | 0.792 | 1.000 | 0.750 | 0.708 | 0.875 | 0.435 | 0.818 | 0.833 |
| Cpa 114 | A | 14 | 17 | 14 | 8 | 15 | 15 | 17 | 13 | 9 |
|  | *H*E | 0.931 | 0.933 | 0.916 | 0.848 | 0.924 | 0.919 | 0.927 | 0.872 | 0.874 |
|  | *H*O | 0.917 | 0.958 | 0.875 | 0.789 | 0.792 | 0.917 | 0.875 | 0.833 | 0.900 |
| Cha 17 | A | 11 | 16 | 9 | 16 | 12 | 12 | 14 | 17 | 14 |
|  | *H*E | 0.855 | 0.925 | 0.750 | 0.928 | 0.879 | 0.900 | 0.911 | 0.932 | 0.925 |
|  | *H*O | 1.000* | 1.000 | 1.000** | 1.000 | 1.000* | 1.000 | 1.000 | 1.000 | 1.000 |
| Cha 20 | A | 8 | 6 | 11 | 10 | 9 | 8 | 11 | 7 | 8 |
|  | *H*E | 0.733 | 0.780 | 0.890 | 0.848 | 0.762 | 0.822 | 0.909 | 0.809 | 0.870 |
|  | *H*O | 1.000** | 1.000** | 1.000 | 1.000* | 1.000** | 1.000* | 1.000 | 1.000** | 1.000* |
| Mean | A | 7.6 | 9.2 | 9.6 | 9.1 | 9.9 | 9.7 | 9.9 | 10.3 | 10.8 |
|  | *H*E | 0.672 | 0.736 | 0.768 | 0.807 | 0.796 | 0.773 | 0.743 | 0.811 | 0.792 |
|  | *H*O | 0.706 | 0.727 | 0.743 | 0.786 | 0.783 | 0.735 | 0.714 | 0.742 | 0.721 |

Sample size (*n*), number of allele (*A*), expected heterozygosity (*H*E) and observed heterozygosity (*H*O). Significant deviations from Hardy-Weinberg genotype ratios: * *P*<0.05, ** *P*<0.01, none remain significant after sequential Bonferroni correction.
